# Rapid Removal of Zinc(II) from Aqueous Solutions Using a Mesoporous Activated Carbon Prepared from Agricultural Waste

**DOI:** 10.3390/ma10091002

**Published:** 2017-08-28

**Authors:** Xiaotao Zhang, Yinan Hao, Ximing Wang, Zhangjing Chen

**Affiliations:** 1 College of Science, Inner Mongolia Agricultural University, Hohhot 010018, China; lianzixiaotao@163.com; 2 College of Material Science and Art Design, Inner Mongolia Agricultural University, Hohhot 010018, China; nanyihao83@163.com; 3 Department of Sustainable Biomaterials Virginia Tech University, Blacksburg, VA 24061, USA; chengo@vt.edu

**Keywords:** activated carbon, adsorption, zinc(II), desorption, mechanism

## Abstract

A low-cost activated carbon (XSBLAC) prepared from *Xanthoceras* *Sorbifolia* *Bunge* *hull* via chemical activation was investigated to determine its adsorption and desorption properties for zinc(II) ions from aqueous solutions. XSBLAC was characterized based on its N_2_-adsorption/desorption isotherm, EDX, XRD, SEM and FTIR results. An adsorption study was conducted in a series of experiments to optimize the process variables for zinc(II) removal using XSBLAC. Modeling the adsorption kinetics indicated good agreement between the experimental data and the pseudo-second-order kinetic model. The Langmuir equilibrium isotherm fit the experimental data reasonably well. The calculated enthalpy (Δ*H*^0^), entropy (Δ*S*^0^) and Gibbs free energy (Δ*G*^0^) values revealed the endothermic and spontaneous nature of the adsorption process. HNO_3_ displayed the best desorption performance. The adsorption mechanism was investigated in detail through FTIR and SEM/EDX spectroscopic analyses. The results suggested that XSBLAC is a potential biosorbent for removing zinc(II) from aqueous solutions.

## 1. Introduction

Zinc(II) is frequently found in effluents discharged from industries, such as electroplating, pigments, battery manufacturing units, mining, metallurgy and municipal wastewater treatment plants. Zinc(II) is a well-known toxic metal ion and can threaten human life by bioaccumulating in the food chain. The World Health Organization recommended a maximum acceptable zinc concentration in drinking water of 5.0 mg/L [1]. The removal of zinc(II) ions or decreasing its concentration to the permitted levels before discharge is necessary to prevent deleterious effects on the ecosystem and public health [2].

Numerous methods have been employed to remove zinc(II) ions from wastewater, including precipitation, coagulation, ion exchange, membrane filtration, and electrolysis [3,4,5,6,7]. The cost and the generation of harmful wastes are important parameters relevant to most of these processes, especially for low concentrations of zinc(II). Currently, adsorption is regarded as a powerful technology for removing zinc(II) from aqueous solutions. Commercial activated carbon (AC) has been studied as an adsorbent for wastewater treatment; it has highly developed porosity, a large internal surface area, and relatively high mechanical strength [8]. However, manufacturing AC is very expensive [9]. To overcome this disadvantage, AC can be produced from a wide variety of raw and low-cost agricultural waste byproducts, including coconut shell, pecan shell, rice husk, oil palm shell, sugarcane bagasse, palm shell, and sawdust [10,11,12,13,14,15,16,17].

The production of AC from agricultural by-products is an increasingly interesting research field because it addresses the problem of the disposal of agro-residues. As the major source of energy tree species, *Xanthoceras Sorbifolia Bunge* has been widely and extensively cultivated in northern areas, especially the Inner Mongolia autonomous region of China, which has played a very significant role in the agricultural industry. Moreover, the Inner Mongolia region owns lots of processing industrial enterprises, which often produce a number of pollutants containing heavy metals. Therefore, we could efficiently utilize the biomass resources of *Xanthoceras sorbifolia Bunge*, preparing it as a potential high adsorbent and applying it to industrial wastewater for the purpose of “waste to manage waste”. It is an attractive method to solve issues related to the sustainable development of industrialization in the north of China. From this point of view, the way in which agriculture is combined with industry of the local region to manage the ecological environment is the novelty of this work. In practice, *Xanthoceras Sorbifolia Bunge hull* (XSBL) is a by-product of production. XSBL is available in large quantities at no cost and could be a good basis for the development of adsorbent materials. Currently, the existing literature contains no information regarding the removal of zinc(II) using *Xanthoceras Sorbifolia Bunge hull* AC (XSBLAC). 

The present work aims to prepare AC from XSBL via chemical activation using H_3_PO_4_ and characterize its ability to treat zinc(II)-containing wastewater. This XSBLAC production process is novel because it adopts the National Invention Patent method [18]. The properties of XSBLAC are investigated by analyzing N_2_ adsorption/desorption, energy dispersive spectrometry (EDX), X-ray diffraction (XRD), scanning electron microscopy (SEM) and Fourier transform infrared spectroscopy (FTIR) results. Furthermore, the adsorption and desorption capacities of zinc(II) are observed in detail. Each factor influencing the adsorption and desorption behavior of XSBLAC, including the initial concentration of zinc(II), pH value, adsorption temperature, adsorption time, HNO_3_ concentration, desorption temperature and desorption time, is systematically studied using various scenarios. The adsorption kinetics and isotherms on XSBLAC are determined; the enthalpy (Δ*H*^0^), entropy (Δ*S*^0^) and Gibbs free energy (Δ*G*^0^) values are calculated; and the mechanism of zinc(II) adsorption is discussed based on Fourier transform infrared (FTIR) and SEM/energy-dispersive X-ray (EDX) spectroscopic analyses. Finally, exploratory research results regarding the recycling application of XSBLAC offer a reference for zinc(II) removal, and its regeneration ability is evaluated after five adsorption/desorption cycles, showing good recycling ability. 

## 2. Experiments

### 2.1. Materials

The XSBL used in this study as a raw material was collected from Chifeng, China. Prior to use, the sample was washed with hot deionized water to remove dirt particles, dried in the sun for a specific period of time, ground in a high-speed rotary cutting mill, and screened to a particle size of 0.5–0.8 mm for experimental use. A stock solution of zinc(II) (1000 mg/L) was prepared using analytical reagent (A.R.)-grade nitrate salts purchased from Tianjin Beilian Fine Chemicals Co., Ltd. (Tianjin, China). All other chemicals were reagent grade and were used without further purification. All solutions were prepared using deionized water.

### 2.2. AC Preparation

Solid XSBL residue was washed to neutrality using deionized water and dried at 100 °C for 24 h in a hot air oven (Memmert VO400, Schwabach, Germany). Subsequently, the dried mass was soaked in H_3_PO_4_ (85 wt %) and magnetically stirred (500 rpm) for 1 h. The impregnation ratio was calculated as the ratio of the weight of H_3_PO_4_ in solution to the weight of the XSBLBL used. The material was carbonized at 500 °C in a tube furnace (FSX2-12-15N, Tianjin, China) for 4 h. The sample was then cooled to room temperature in a nitrogen atmosphere and washed with deionized water until the pH level stabilized. It was dried in an oven overnight at 120 °C, ground, and finally sieved to 200 mesh [18] using standard sieves (Model Φ200). Then, this carbon powder was stored in an airtight packet for experimental use.

### 2.3. Adsorption Studies

An amount of XSBLAC 0.1000 g (BS210S, Sartorius, Gottingen, Germany) was accurately weighed and added to 50 mL of zinc(II) solution with a known concentration. The suspension was stirred at a uniform speed of 120 rpm in a thermostatic shaker (SHA-C, Zhangjiagang, China), and its pH was adjusted using a certain amount of NaAc-HAc solution with a pH meter (PB-10, Sartorius, Germany). When the adsorption equilibrium was reached, the mixture was centrifuged at 6000 rpm for 10 min (H2050R, Changsha, China). The residual concentration of zinc(II) in the supernatant was determined using an ultraviolet (UV)-Visible spectrophotometer (TU-1901, Beijing, China). The adsorption experiments were conducted with different initial zinc(II) concentrations, pH values, adsorption temperatures and times. Considering the experimental errors, three experiments were run in parallel under the same conditions, and the obtained results were based on the average values. The adsorption capacity of the zinc(II) solution was determined using Equation (1) [19]:

(1) $$q_{t,1}=\frac{(C_{0}-C_{t})V_{1}\times 65.38}{m_{1}}$$

where *q_t_*_,1_ (mg/g) is the adsorption capacity at time *t* (min). *C*_0_ and *C_t_*_,1_ (mol/L) refer to the initial zinc(II) concentration and the final concentration at time *t* (min), respectively. *V*_1_ (mL) refers to the volume of zinc(II), and *m*_1_ (g) is the mass of the adsorbent. In the calculation *q_t_*_,1_, no losses of zinc(II) to any other mechanisms (e.g., volatilization, sorption on glassware, or degradation) were assumed.

### 2.4. Desorption and Regeneration Studies

Samples (0.1000 g) of zinc(II)-loaded XSBLAC were accurately weighed and transferred into 50-mL HNO_3_ solutions with different concentrations. Each mixture was placed in an ultrasonic cleaning machine (KS-300EI, Ninbo, China). When equilibrium was reached at a certain temperature, the suspension was centrifuged, and the desorbed zinc(II) concentrations in the solution were determined. Considering the experimental errors, three experiments were performed, and the reproducibility of the results was within ±3%. The desorption capacity of the zinc(II)-loaded XSBLAC was calculated according to Equation (2) [20].

(2) $$q_{t,2}=\frac{C_{t,2}V_{2}\times 65.38}{m_{2}}$$

where *q_t_*_,2_ (mg/g) refers to the desorption amount at time *t* (min). *C_t_*_,2_ (mol/L) is the zinc(II) concentration in the desorbed solution at time *t* (min). *V*_2_ (mL) refers to the total volume of the desorption solution, and *m*_2_ (g) is the mass of the zinc(II)-loaded XSBLAC.

Repeated batch experiments were performed to examine the reusability of XSBLAC for zinc(II) removal. The material was washed with deionized water to remove the remaining acid and dried in an oven (DZF-6210, Shanghai, China) at 120 °C for subsequent zinc(II) adsorption. The zinc(II)-adsorption and desorption capacities were determined and analyzed. The adsorption/desorption process was performed consecutively five times.

## 3. Results and Discussion

### 3.1. Adsorbent Characterization

The specific surface area and porous system of the XSBLAC were characterized based on N_2_-adsorption/desorption isotherms (Micromeritics ASAP 2020, Norcross, GA, USA). XRD analysis of the powdered samples was performed using an X-ray power diffractometer with a Cu anode (PAN Alytical Co., X’pert PRO, Almelo, The Netherlands) at 40 kV and 40 mA and scanning from 4° to 18° at 8°/min. The morphological changes and surface analyses of the samples were conducted using SEM-EDX (HITACHI S-4800, Tokyo, Japan). FTIR spectra were recorded in KBr pellets with a FTIR spectrophotometer (Thermo Nicolet, NEXUS, TM, Waltham, MA, USA).

### 3.2. Properties of XSBLAC

N_2_-adsorption/desorption isotherms provide qualitative information regarding the porosity of carbonaceous adsorbents [21]. The surface physical parameters of XSBLAC obtained from its N_2_-adsorption/desorption isotherm are summarized in Table 1. From the results, the highest surface area (688.62 m^2^/g) and total pore volume (0.377 cm^3^/g) of XSBLAC were calculated using the t-plot method. Additionally, the structure of XSBLAC was found to be loose, with many pores generated on its surface. According to the International Union of Pure and Applied Chemistry (IUPAC), the mesopore structure of XSBLAC can be supported by its average pore diameter (D*_p_* = 2.2 nm).

**Figure 1:**
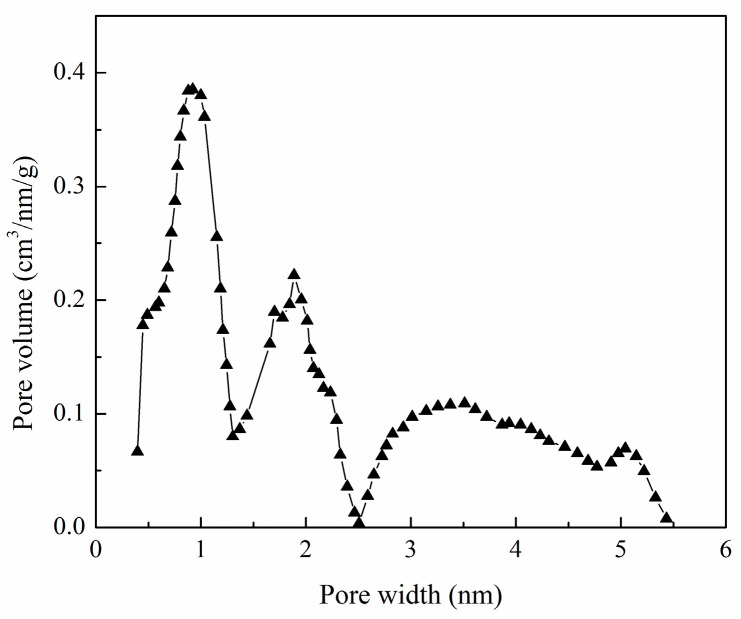
Pore size distribution of XSBLAC.

Figure 1 shows the pore size distribution of XSBLAC. XSBLAC has pore sizes between 0.5 nm and 5.5 nm and displays a wide pore size distribution with a low pore volume. From Table 2, it can be seen that the mesopore surface increased remarkably. Thus, a significant amount of micropores became mesopores. 

Elemental analysis was conducted for XSBLAC and XSBL by EDX. As indicated in Table 2, the C, O, N, Cl and Na contents for XSBLAC were as follows: 80.26%, 12.92%, 5.62%, 0.75% and 0.45%, respectively. Additionally, XSBLAC prepared from XSBL using the activating reagent H_3_PO_4_ during the treatment process showed a higher C content (80.26%) than XSBL (60.33%). 

**Figure 2:**
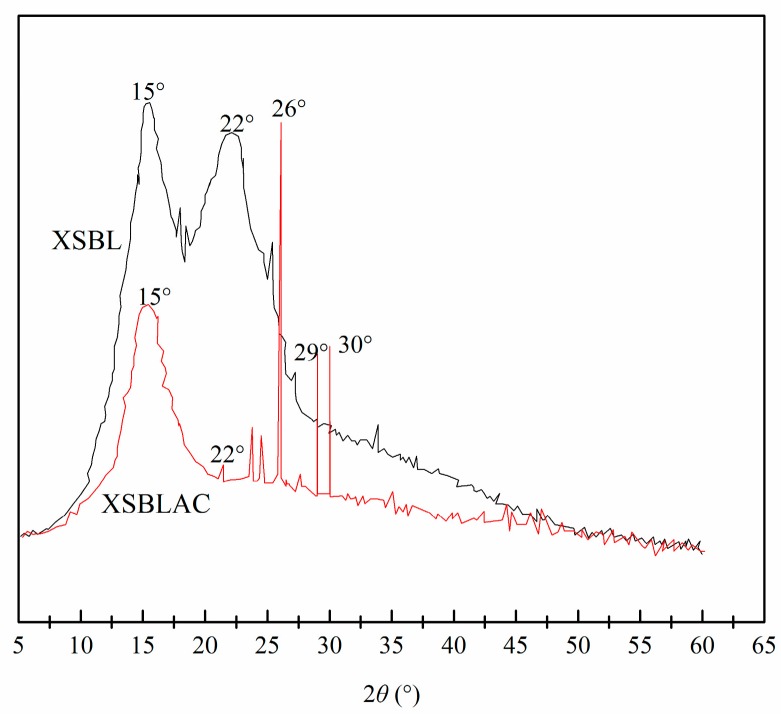
XRD patterns of raw XSBL and XSBLAC.

XRD is an effective method for determining the morphological features of adsorbents. Figure 2 shows the XRD images of XSBL and the prepared XSBLAC. Two broad peaks are present in the XRD of the precursor, observed near 2*θ* = 15° and 22° and attributed to the aliphatic chain of XSBLBL; this indicates that XSBLBL has a turbostratic structure and defined amorphous carbons. The XRD pattern of XSBLAC (Figure 2) shows diffraction peaks at 2*θ* = 26°, 29° and 30°, likely because of internal etching during H_3_PO_4_ activation and increased numbers of complex pores in XSBLAC and shaped graphite-like microcrystalline layers, suggesting the formation of more amorphous carbon-based graphite microcrystallites [22]. Additionally, the dispersion peak (2*θ* = 15°) persisted, illustrating that XSBLAC existed in a type of amorphous state.

**Figure 3:**
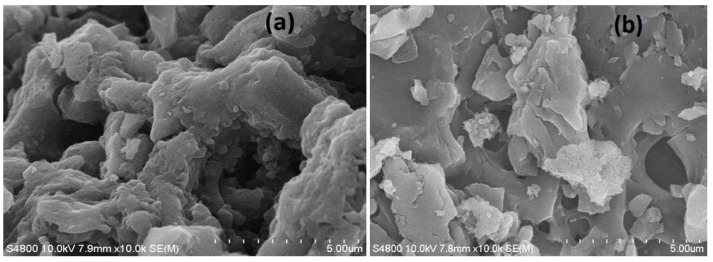
SEM image of raw XSBL (**a**) and XSBLAC (**b**).

The SEM images of XSBL and XSBLAC are presented in Figure 3. This figure shows that the surface of XSBL contains many thin sheets or layers with large pores in its structure (Figure 3a). However, the AC appeared to have a well-developed coarse porous surface with irregular pores and breaks (Figure 3b). Mixtures of micropores and mesopores are present in the SEM images. This result may be attributed to the creation of pores and the substantial removal of inorganic compounds in the raw structure during activation, eventually yielding a porous surface.

### 3.3. Effect of pH Value on Adsorption

**Figure 4:**
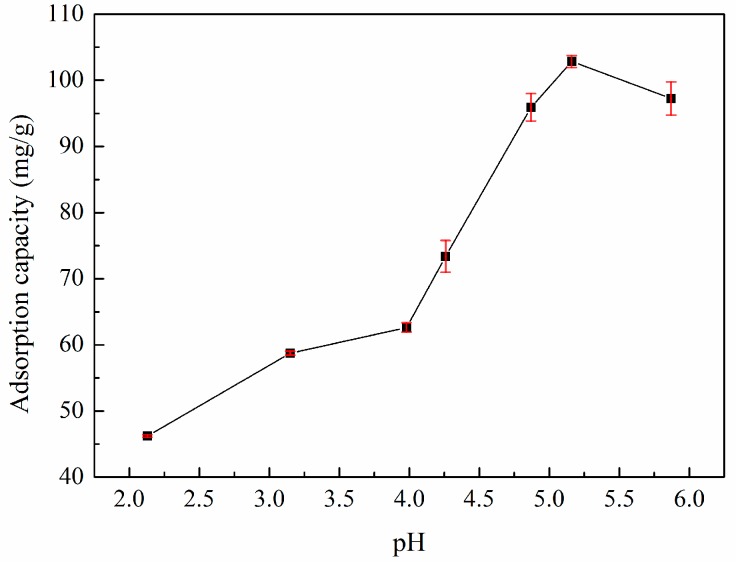
Effect of the pH values on the adsorption capacity of XSBLAC for zinc(II). (Adsorption experiments-sample dosage: 0.05 g; initial zinc(II) concentration: 0.015 mol/L; pH range: 2.0–6.0; temperature: 60 °C; adsorption time: 40 min).

The pH value of the zinc(II) solution is highly influential and must be considered during the adsorption process. Therefore, experiments were initially performed to optimize the pH value, testing values from 2.0 to 6.0, to investigate the effect of pH on zinc(II) removal, as shown in Figure 4. The adsorption of zinc(II) by XSBLAC was highly pH dependent because the superficial charge of XSBLAC is affected by the pH value [23]. Indeed, the maximum adsorption capacity for zinc(II) was achieved at a pH value of 5.2. This result can be explained by the competition between H^+^ and zinc(II) ions for activated adsorption sites on the XSBLAC surface at low pH levels; when the pH increases, the covered H^+^ leaves the AC surface, making more adsorption sites available for zinc(II). According to the solubility product constant of zinc(II) hydrolysis (p*K*_sp_*^θ^* = 16.92), as the pH value increases, the negative charge density also increases, resulting in the production of oxygen-containing functional groups and/or ligands in XSBLAC. Therefore, all pH values used were below 6.0, ensuring that no hydroxide precipitation occurred during the adsorption process. Similar pH effects on the adsorption of heavy metals have been reported in the literature [24,25]. Hence, the optimal pH value for zinc(II) adsorption in this study was 5.2.

### 3.4. Effect of Temperature on Adsorption

**Figure 5:**
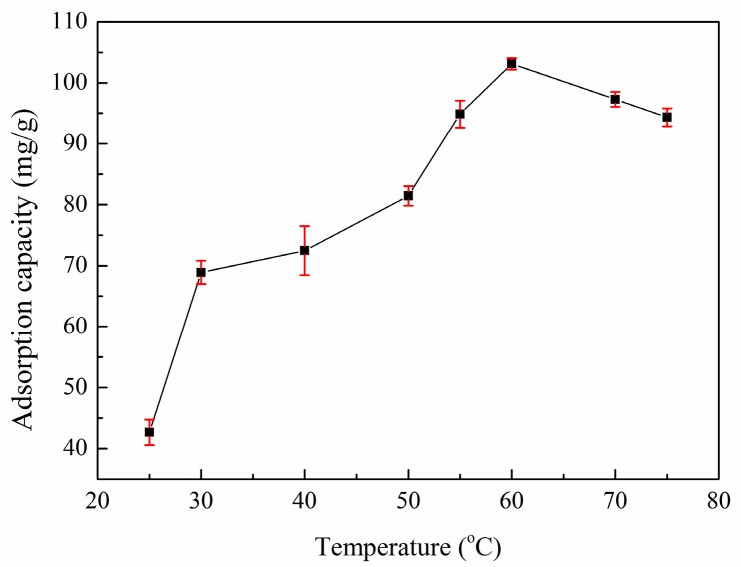
Effect of temperature on the adsorption capacity of XSBLAC for zinc(II). (Adsorption experiments-sample dosage: 0.05 g; initial zinc(II) concentration: 0.015 mol/L; pH range: 5.16; temperature: 25–75 °C; adsorption time: 40 min).

It is important to investigate the effect of temperature on zinc(II) adsorption for practical applications. Here, the relationship between the temperature and the adsorption capacity of XSBLAC is discussed under isothermal conditions at different temperatures, as shown in Figure 5. The adsorption capacity of XSBLAC increased as the temperature increased from 25 to 60 °C, indicating that high temperatures facilitate the adsorption of zinc(II) on the surface of XSBLAC. This result can be attributed to the fact that high temperature may produce a loosening effect within the structure and activate the functional groups of XSBLAC, leading to further adsorption of zinc(II) ions into the structure of AC. However, at temperatures above 60 °C, the adsorption capacity decreased, accounting for the endothermic behavior of the adsorption process. These results can be attributed to the chemical adsorption action between XSBLAC and zinc(II). In the following experiments, a pH of 5.2 was selected as the optimum pH value.

### 3.5. Adsorption Kinetics

**Figure 6:**
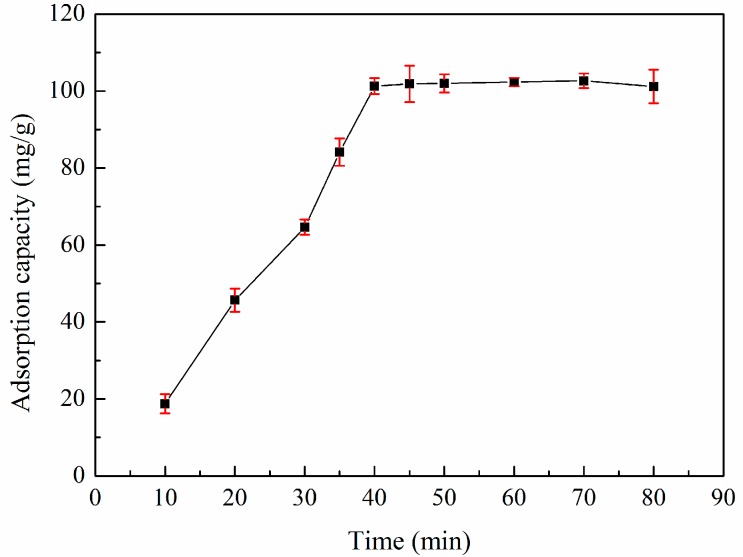
Effect of time on the adsorption capacity of XSBLAC for zinc(II). (Adsorption experiments-sample dosage: 0.05 g; initial zinc(II) concentration: 0.015 mol/L; pH value: 5.16; temperature: 60 °C; adsorption time: 10–80 min).

The effect of adsorption time on the removal of zinc(II) by XSBLAC was investigated, and the results are shown in Figure 6. The adsorption capacity of XSBLAC greatly increased from 10 to 40 min until equilibrium and then remained nearly stable as the adsorption time increased further. This behavior may be why zinc(II) ions first adsorbed on the surface’s unsaturated activated functional sites, subsequently diffused into the XSBLAC’s micropores, and finally adsorbed on functional sites, thereby saturating the material’s mesopores and reaching adsorption equilibrium. Therefore, an adsorption time of 40 min was chosen as the optimal equilibrium time for the adsorption of zinc(II) on XSBLAC under the experimental conditions.

To study the potential rate-controlling steps of adsorption, pseudo-first-order, pseudo-second-order, intraparticle diffusion, Elovich and Bangham kinetic models are generally used to fit the experimental data, which are described as Equations (3)–(7) [26,27,28].

(3) $$\mathrm{lg}(q_{e}-q_{t,1})=\mathrm{lg}q_{e}-\frac{k_{1}t}{2.303}$$

(4) $$\frac{t}{q_{t,1}}=\frac{1}{k_{2}q_{e}{}^{2}}+\frac{t}{q_{e}}$$

(5) $$q_{t}=k_{i}t^{0.5}$$

(6) $$q_{t}=\frac{1}{\beta }\ln(\alpha \beta )+\frac{1}{\beta }\ln t$$

(7) $$\log q_{t}=\log k_{r}+\frac{1}{m}\log t$$

where *q_e_* and *q_t_*_,1_ are the amounts of zinc(II) ions adsorbed (mg/g) at equilibrium and at time *t* (min), respectively; *k*_1_ (min^−1^) is the pseudo-first-order rate constant; *k*_2_ [g·(mg/min)^−1^] is the rate constant of the pseudo-second-order adsorption kinetic equation; *k_i_* [mg·(g·min^0.5^)^−1^] is an intraparticle diffusion rate constant; *α* [mg·(g·min)^−1^] is an initial adsorption rate; and *β* (g/mg) is related to the surface coverage and activation energy for chemisorption; *k_r_* [mg·g^−1^·min^−1^] is the rate constant of adsorption; 1/*m* is an indicator of the adsorption intensity.

**Figure 7:**
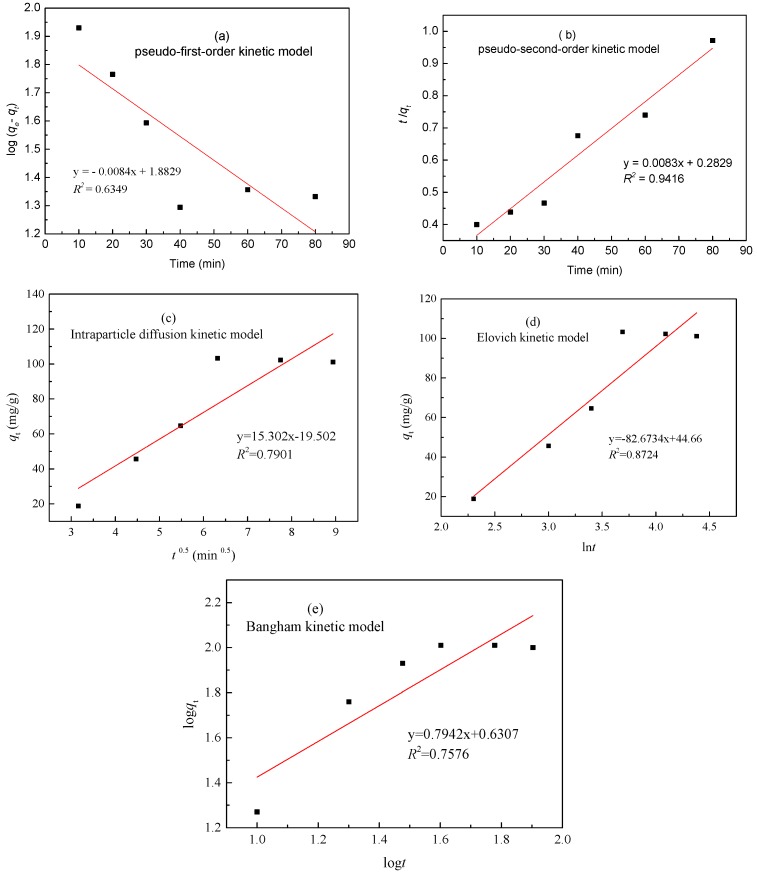
Pseudo-first-order (**a**); pseudo-second-order (**b**); intraparticle diffusion (**c**); Elovich (**d**) and Bangham (**e**) adsorption kinetic equations fitting curves of experimental data. (Adsorption experiments-sample dosage: 0.05 g; initial zinc(II) concentration: 0.015 mol/L; pH value: 5.16; temperature: 60 °C).

The fits of these five models were checked by each linear plot of ln(*q_e_* − *q_t_*) versus *t* (Figure 7a), (*t*/*q_t_*) versus *t* (Figure 7b), *q_t_* versus *t*^0.5^ (Figure 7c), *q_t_* versus ln*t* (Figure 7d), and ln*q_t_* verse ln*t* (Figure 7e), respectively. The *R*^2^ and constant values for the five adsorption kinetic models were calculated and are given in Table 3. According to the calculated kinetic model parameters in Table 3 and from comparing the experimental equilibrium adsorption capacity, it was clearly found that the calculated *q_e_* value (103.82 mg/g) agreed with the experimental data (110.48 mg/g), and the pseudo-second-order model produced a good fit based on the value of *R*^2^ (0.9416), indicating that the pseudo-second-order kinetics model can reflect the adsorption process of XSBLAC. Therefore, it is obvious that chemical adsorption should be the rate limiting step of the adsorption of zinc(II) ions onto the prepared activated carbon XSBLAC.

### 3.6. Adsorption Isotherms

**Figure 8:**
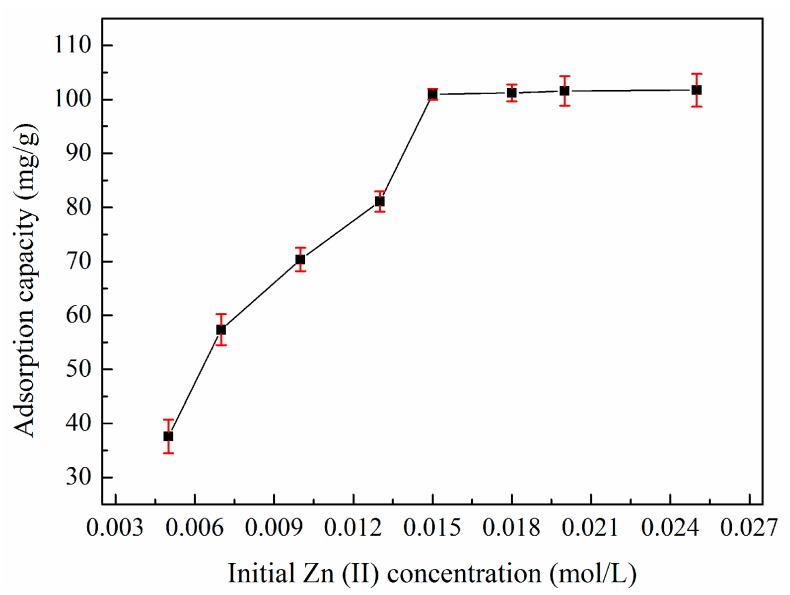
Effect of initial zinc(II) concentration on the adsorption capacity of XSBLAC for zinc(II). (Adsorption experiments-sample dosage: 0.05 g; initial zinc(II) concentration: 0.005–0.025 mol/L; pH value: 5.16; temperature: 60 °C; adsorption time: 40 min).

The effect of the initial zinc(II) concentration on the adsorption capacity of XSBLAC is shown in Figure 8. The initial concentration of zinc(II) substantially influenced the adsorption capacity, which increased sharply as the initial zinc(II) concentration increased until equilibrium but hardly increased as the zinc(II) concentration was increased further. Thus, the zinc(II)-removal process probably involves an interaction between the activated sites on XSBLAC and zinc(II), which may result from the increase in initial zinc(II) concentration accelerating the bonding of zinc(II) onto XSBLAC, thereby increasing the driving force underlying the concentration gradient [29]. Increasing the zinc(II) concentration further did not increase the adsorption capacity because the activated sites on XSBLAC were saturated. An initial zinc(II) concentration of 0.015 mol/L was used to ensure that the adsorption equilibrium would be reached.

Adsorption isotherm models are commonly used to describe the characteristics of an adsorption process between solid and liquid phases when adsorption equilibrium is reached. The linear forms of the Langmuir and Freundlich isotherms are represented, respectively, by Equations (8) and (9) [30]:

(8) $$\frac{C_{e}}{q_{e}}=\frac{1}{bq_{m}}+\frac{C_{e}}{q_{m}}$$

(9) $$\ln q_{e}=\ln k_{f}+\frac{1}{n}\ln C_{e}$$

where *C_e_* is the concentration of zinc(II) at equilibrium (mol/L), *q_e_* is the amount adsorbed (mg/g), *q_m_* is the complete monolayer adsorption capacity (mg/g), *b* is the Langmuir constant related to the adsorption capacity (L/mg), and *n* and *k_f_* are the Freundlich constants.

**Figure 9:**
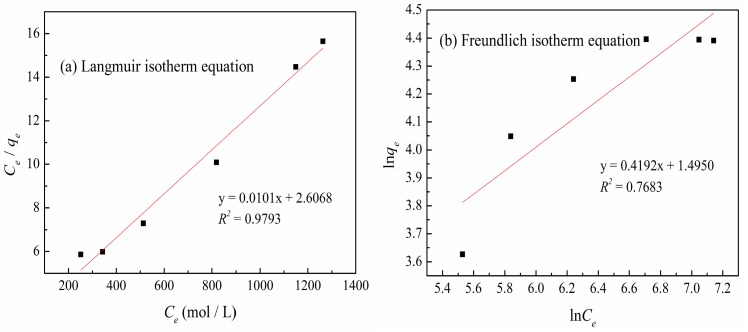
Langmuir (**a**) and Freundlich (**b**) isotherm equations fitting curves of the experimental data. (Adsorption experiments-sample dosage: 0.05 g; pH value: 5.16; temperature: 60 °C; adsorption time: 40 min).

A plot of *C_e_*/*q_t_* versus *C_e_* is shown in Figure 9a, and the Langmuir constants were calculated using the slopes and intercepts listed in Table 4. Similarly, a plot of ln*q_e_* versus ln*C_e_* (Figure 9b) was used to evaluate the Freundlich constants, which are also presented in Table 3. From the correlation coefficient (*R*^2^) values in Table 4, *R*^2^ value of 0.9793 (*p* (1.327 × 10^−4^) < 0.01) for the Langmuir isotherm adsorption model is higher than that for the Freundlich model of *R*^2^ value of 0.7683 (*p* (0.0026) < 0.01), and there was no significant in the adsorption of zinc(II) ions. The experimental data for the adsorption process had better correlation coefficients values and better fits with the Langmuir isotherm model than with the Freundlich model (Figure 9). Therefore, the adsorption process of zinc(II) onto XSBLAC was found to follow the Langmuir isotherm model (*R*^2^ = 0.9793, *p* < 0.01), with a maximum monolayer adsorption capacity of 103.82 mg/g. This result revealed the anchoring of zinc(II) to the abundant functional groups on XSBLAC with the formation of monolayer surface coverage that was homogenous in nature. Moreover, the characteristics of the Langmuir equation can be explained in terms of the equilibrium parameter (*R_L_*) according to Equation (10) [31]:

(10) $$R_{L}=\frac{1}{1+bC_{0}}$$

where *b* is the Langmuir constant (L/mg), and *C*_0_ is the initial concentration (mol/L). The value of *R_L_* describes the nature of the adsorption: irreversible (*R_L_* = 0); favorable (0 < *R_L_* < 1); linear (*R_L_* = 1); unfavorable (*R_L_* > 1) [32]. In this study, the *R_L_* value over the given initial zinc(II) concentration range was calculated (0.1362) and is shown in Table 4, confirming that XSBLAC was favorable for zinc(II) adsorption under the adsorption conditions employed.

Thermodynamic parameters were calculated to determine which process would occur spontaneously. The variation of the thermodynamic parameters (Δ*H*^0^, Δ*S*^0^ and Δ*G*^0^) should provide insight into the mechanism and adsorption of an isolated system [33]. The enthalpy (Δ*H*^0^) and entropy (Δ*S*^0^) values can be calculated from the slope and intercept of the plot of ln*K* vs. 1/*T* according to Equation (11) [34]:

(11) $$\ln K=\frac{\Delta S^{0}}{R}-\frac{\Delta H^{0}}{RT}$$

where *R* (8.314 J/mol·K) is the universal gas constant, and *T* (K) is the adsorption temperature in Kelvin. The Gibb’s free energy change (Δ*G*^0^) was calculated from the Langmuir equilibrium constant in units of liters per mole according to Equation (12) [35]:

(12) $$\Delta G^{0}=-RT\ln K$$

where *K* (L/mol) is from the Langmuir equation and has units of liters per mole. The Gibb’s free energy (Δ*G*^0^) was −19.91 KJ/mol at 60 °C, and its negative values verified the thermodynamic feasibility and spontaneity of the adsorption process under the applied experimental conditions. Δ*H*^0^ was 59.68 KJ/mol, which further confirmed the endothermic behavior of XSBLAC. The positive value of Δ*S*^0^ (0.239 J/K/mol) may be attributed to the affinity of XSBLAC for zinc(II) and the increasing randomness at the solid-liquid interface. It can be concluded that the adsorption of XSBLAC was an endothermic and spontaneous process based on the thermodynamic data of the adsorption isotherm.

Many thermodynamic properties of polymer-solutions such as solubilities, swelling equilibria, and the colligative properties can be expressed in terms of the polymer-solvent interaction parameter. Generally, the Flory–Huggins interaction parameter describes the degree of segregation in polymer blends and block copolymers, which is typically expressed as a function of temperature, *T*. To establish a relationship between *χ* and *T*, we follow the approach of Rana et al. and calculate using Equations (13)–(15) [36,37,38].

(13) $$\Delta G^{0}=\Delta H^{0}-T\Delta S^{0}$$

(14) $$\Delta G^{0}=RT(\frac{\varphi_{1}}{N_{1}}\ln\varphi_{1}+\frac{\varphi_{2}}{N_{2}}\ln\varphi_{2}+\chi \varphi_{1}\varphi_{2})$$

(15) $$\chi =\frac{\alpha }{T}+\beta$$

where *ϕ*_1_ is the volume fraction of XSBLAC particles, *ϕ*_2_ is the volume fraction of zinc(II), *N*_1_ is the molecular volume of XSBLAC particles, *N*_2_ is the molecular volume of zinc(II), *χ* is the F-H interaction parameter, *T* is the temperature, *α* and *β* are parameters for enthalpic and entropic contributions to *χ*, respectively. 

*χ* has been found to decrease with temperature with a dependence that is approximately linear with, but not proportional to 1/*T* for many systems. In this work, the *χ* with a value of 0.34 is extrapolated and obtained, which is representative of a miscible system and an exothermic heat of XSBLAC and zinc(II). It should be noted that the *χ* value is very close to zero, suggesting more favorable mixing at the temperature of 60 °C, which more conducive to the adsorption process between XSBLAC and zinc(II).

### 3.7. Desorption and Regeneration

**Figure 10:**
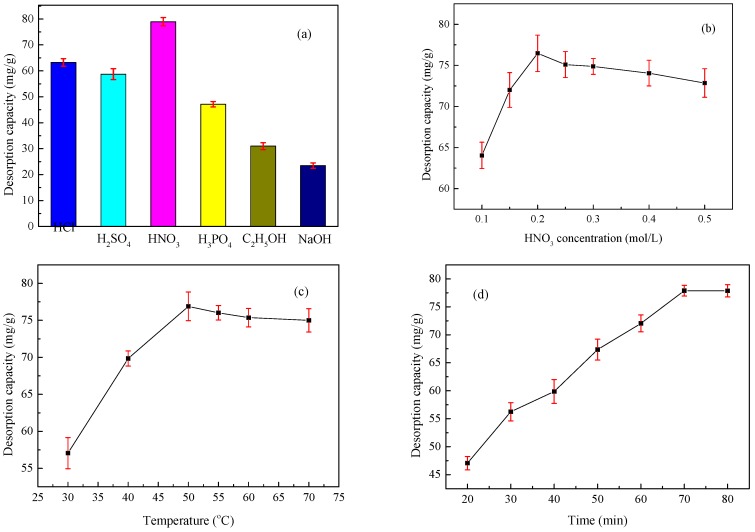
Effects of various desorption reagents (**a**); HNO_3_ concentration (**b**); temperature (**c**) and time (**d**) on desorption capacity of XSBLAC for zinc(II).

Performing desorption and regeneration studies of spent adsorbents can produce various benefits, such as lowering the cost associated with the adsorption process, recovering valuable zinc(II), and reducing possible secondary pollution. Thus, a desorbing solution must be inexpensive, effective, and non-polluting. Batches of desorption and regeneration experiments were conducted in the present work using HCl, H_2_SO_4_, HNO_3_, H_3_PO_4_, C_2_H_5_OH and NaOH as the desorbing eluent to test their effects on desorption (Figure 10a). Figure 10a clearly shows that NaOH is almost ineffective at releasing bonded zinc(II) ions from XSBLAC. The desorption efficiency of C_2_H_5_OH was slightly higher than that of NaOH but still showed weak potential for detaching zinc(II) compared with acid solutions. Among the four acidic desorption solutions, HCl, H_2_SO_4_, HNO_3_ and H_3_PO_4_, and HNO_3_ were found to be good reagents for the regeneration of zinc(II)-loaded XSBLAC. This result was expected because under acidic conditions, electrostatic interactions occurred on the surface of XSBLAC, which was became protonated by H^+^ ions, thereby allowing the desorption of positively charged zinc(II). The desorption efficiency of HNO_3_ was better than those of HCl, H_2_SO_4_ and H_3_PO_4_ for zinc(II) removal from XSBLAC.

The effect of using HNO_3_ solution as a desorption reagent on the desorption capacity of zinc(II)-loaded XSBLAC, including the HNO_3_ concentration, desorption temperature and ultrasonic desorption time, is discussed in detail. The desorption capacity initially increased and then decreased with increasing HNO_3_ concentration (Figure 10b), possibly because the accumulated H^+^ concentration increases the concentration gradients of zinc(II) and H^+^, which constitute the driving force underlying ion exchange and favoring the desorption process [39]. The desorption capacity first increased and then slightly decreased with increasing desorption temperature (Figure 10c); this behavior is likely attributable to the higher temperature, which may enhance the efficiency and activity of the adsorption sites of XSBLAC until equilibrium is reached [40]. The desorption capacity initially increased and then remained constant with increasing ultrasonic desorption time (Figure 10d), consistent with the production of holes, and then reached saturation under ultrasonic conditions [41]. Subsequently, the effect of regenerating and reusing the XSBLAC for five consecutive adsorption/desorption cycles of zinc(II) was investigated, and the results are listed in Table 5. The zinc(II)-adsorption capacity decreased only slightly with increasing reuse times, and the desorbed XSBLAC was effective for re-adsorption in the fourth cycle under the experimental conditions used. Thus, XSBLAC presents excellent reusability and can be employed as a suitable reagent for the removal of zinc(II) from wastewater numerous times. 

### 3.8. Adsorption Mechanism

**Figure 11:**
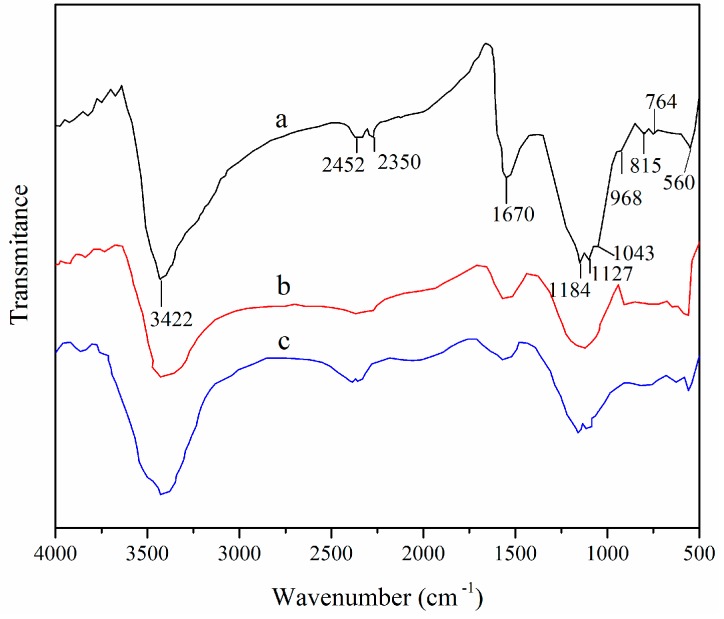
FTIR spectra of XSBLAC (**a**); after adsorption (**b**) and after desorption (**c**) of zinc(II).

The surface functional groups of the adsorbent highly influence its characteristics and adsorption capabilities. The FTIR spectra of XSBLAC after the adsorption and desorption of zinc(II) are presented in Figure 11. The absorbance bands of XSBLAC (Figure 11a) were found to include a broad overlapping band at 3200–3500 cm^−1^, with a peak at 3422 cm^−1^, which can be attributed to the O–H stretching of the hydroxyl groups and intermolecular hydrogen bonds. The position and asymmetry of this band implied the presence of strong hydrogen bonds. This peak shifted to lower wave number 3420 cm^−1^ and weakened after the adsorption of zinc(II) (Figure 11b), indicating that the O–H and the corresponding hydrogen bonds of XSBLAC were broken to react with zinc(II). The bands at 2452 and 2350 cm^−1^ were related to the C–H stretching of methyl groups (Figure 11a) and nearly disappeared after adsorption of zinc(II) ions (Figure 11b). The intensity of the peak at approximately 1670 cm^−1^ (Figure 11a) revealed the characteristics of the C=O stretching in the carboxylic organic acid groups, which were shifted to lowered wave number and greatly reduced after zinc(II) adsorption (Figure 11b). Logically, the peaks appearing at 1184 and 1127 cm^−1^ (Figure 11a) were assigned to P=O groups because H_3_PO_4_ was used as the activating agent, leading to the formation of phosphate acid esters [42]. Furthermore, the band at approximately 1043 cm^−1^ (Figure 11a) was ascribed to the C–O stretching in saturated aliphatic groups, and –C–O and –O–C=O stretching of the carboxyl groups, which was broadened and reduced after adsorption of zinc(II) (Figure 11b) [43]. Adsorption bands that occurred at approximately 968, 815, and 764 cm^−1^ were related to C–H groups of the benzene ring and aromatic alcohols, and the peak at 560 cm^−1^ may be assigned to the outer-of-plane bending of C–O bonds of alcohols; these peaks shrank after adsorption (Figure 11b) [44]. In addition, the FTIR spectrum of XSBLAC after the desorption of zinc(II) ions (Figure 11c) almost coincided with that of the original XSBLAC (Figure 11a). These changes in the absorption peaks indicated that the activated sites on the surface of XSBLAC contained hydroxyl and carboxylic functional groups, and the free carboxyl groups became carboxylates, which suggested during the adsorption reaction between zinc(II) ions and XSBLAC. Generally, ion exchange occurred, and chemical bonds were formed between zinc(II) and the –OH, –C=O, and –O–C=O groups of XSBLAC. Above all, FTIR spectra comparison showed that XSBL-AC may be applicable as an effective renewable adsorber of zinc(II) from wastewater.

**Figure 12:**
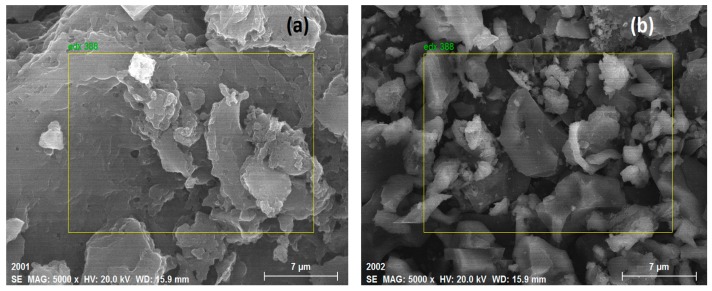
SEM images of XSBLAC before (**a**) and after (**b**) adsorption of zinc(II).

The surface morphologies of XSBLAC before and after zinc(II) adsorption are presented in Figure 12. From Figure 12a, it can be seen that the surface of XSBLAC contained many thin sheets and layers of irregular shapes and sizes with steps and broken edges, which may be an appropriate structure for zinc(II) adsorption. After the adsorption process, the XSBLAC surface was evenly packed with zinc(II) ions and the sheet-stacking structure disappeared (Figure 12b). Distributions of the protruding tips are not evenly spread, hinting that zinc(II) was adsorbed on the activated sites which mainly existed on the protruding tips of the microporous surface, and only selected functional groups are involved in the adsorption of zinc(II) ions. These results indicated that the adsorption of XSBLAC toward zinc(II) may be a chemical interaction, supporting the previously proposed adsorption mechanism. 

**Figure 13:**
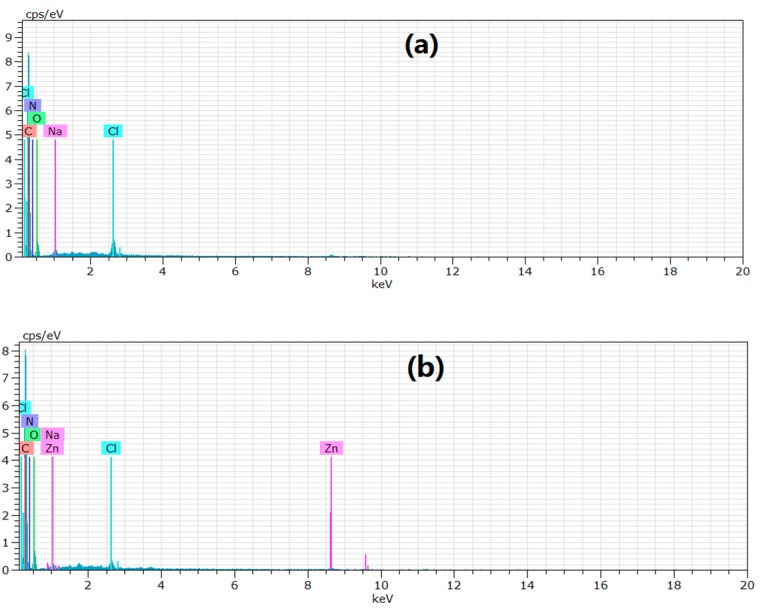
EDX analysis of XSBLAC before (**a**) and after (**b**) adsorption zinc(II).

The EDX is an analytical technique used for elemental analysis. EDX analysis of XSBLAC was performed to confirm existence of zinc(II) on zinc(II)-loaded XSBLAC. The elemental compositions of XSBLAC before and after zinc(II) adsorption were analyzed, and the results are presented in Figure 13. The EDX results of XSBLAC (Figure 13a) revealed the presence of C (80.26%), O (12.92%), N (5.62%), Cl (0.75%) and Na (0.45%). After zinc(II) adsorption, two new peaks of zinc(II) were found in the EDX spectrum (Figure 12b); the at *%* values of the zinc(II)-loaded XSBLAC were C (77.76%), O (10.73%), N (5.21%), Zn (5.88%), Cl (0.31%) and Na (0.11%). This result verified the presence of zinc(II) ions on the surface of XSBLAC after adsorption [45]. From the SEM-EDX results, it was concluded that XSBLAC could to adsorb zinc(II) from wastewater.

### 3.9. Comparison with Previously Reported Data for Zinc(II) Adsorption

Table 6 shows various adsorbents that have been applied in removing zinc(II) from aqueous solution, as reported in the previous literature, for comparison purposes. Through the comparative study, we can conclude that XSBLAC is one of the most powerful adsorbents prepared for industrial wastewater treatment. 

## 4. Conclusions

In summary, a novel XSBLAC was successfully prepared and employed in solution for the removal of zinc(II) ions. The investigated adsorbent was inexpensive and eco-friendly. The experimental results showed that XSBLAC has excellent adsorption capacity for zinc(II) ions from aqueous solutions, which can be attributed to its high surface area (688.62 m^2^/g), total pore volume (0.377 cm^3^/g), O-containing functional activated groups, and electrostatic attraction to zinc(II). Batches of adsorption tests were conducted, and the experimental values obtained were found to be in good agreement with those predicted by the models. The adsorption process was well described by the pseudo-second-order model. The adsorption equilibrium data of zinc(II) on the surface of XSBLAC was suitable for the Langmuir model (maximum monolayer adsorption capacity of 103.82 mg/g), and thermodynamic studies revealed the spontaneous and endothermic nature of the adsorption process. XSBLAC was shown to exhibit an effective desorption performance when washed with an HNO_3_ solution as an eluent, and the maximum desorption capacity obtained was 78.52 mg/g. Additionally, the adsorption capacity after regeneration was even higher until four adsorption/desorption cycles. Furthermore, FTIR and SEM/EDX results demonstrated that zinc(II) ions were mainly adsorbed by selected activated groups in coarse mesopores on the XSBLAC surface; the adsorption mechanism was likely a chemisorption process. Overall, the XSBLAC prepared in this study has potential applications as an alternative and low-cost adsorbent for the removal of zinc(II) ions from aqueous solutions. 

## Figures and Tables

**Table 1:** Pore structure parameters of the sample studied in this work.

Sample	S*_BET_* (m^2^/g)	S*_ext_* (m^2^/g)	S*_ext_*/S*_BET_* (%)	S*_mic_* (m^2^/g)	V*_tot_* (cm^3^/g)	V*_meso_* (cm^3^/g)	V*_meso_*/V*_tot_* (%)	D*_p_* (nm)
XSBLAC	688.62	477.87	69.4	210.43	0.377	0.252	66.8	2.2

Number of analyses: three. S*_BET_*—specific surface area; S*_ext_*—mesopore surface area, S*_ext_*/S*_BET_*—ratio of mesopore surface area to specific surface area, S*_mic_*—micropore surface area, V*_tot_*—total pore volume, V*_meso_*—mesopore volume, V*_meso_*/V*_tot_*—ratio of mesopore volume to total pore volume, D*_p_*—average pore size.

**Table 2:** Global Surface Composition as Determined by EDX Analysis.

Sample	C (at %)	O (at %)	N (at %)	Cl (at %)	Na (at %)	O/C (%)	N/C (%)
XSBLAC	80.26	12.92	5.62	0.75	0.45	16.1	0.07
XSBL	60.33	34.96	3.41	0.93	0.37	60.0	0.06

**Table 3:** Comparison of the estimated adsorption rate constants, *q_e_*, and the correlation coefficients of the five kinetic models.

Metal	*q_e_* (exp) (mg/g)	Parameters	Pseudo-First-Order		Pseudo-First-Order		Intraparticle Diffusion		Elovich		Bangham Model	
Zinc(II)	103.82	*R*^2^	0.6349		0.9416		0.7901		0.8724		0.7576	
		Constants	*k*_1_	0.01329 min^−1^	*K*_2_	2.764 × 10^−4^ min^−1^	*k_i_*	15.302 mg/(g min^0.5^)	*α*	11.2501 mg/(g min)	*K_r_* (mg·g^−1^·min^−1^)	0.7942
			*q_e_* (cal)	76.36 mg/g	*q_e_* (cal)	110.48 mg/g			*β*	0.01327 g/mg	*m*	0.1004

**Table 4:** Adsorption equilibrium constants obtained from the Langmuir and Freundlich isotherms for zinc(II) adsorption onto XSBLAC. (Adsorption experiment sample dosage: 0.05 g; pH value: 5.2; temperature: 60 °C; adsorption time: 40 min).

*q_m_* (mg/g)	Langmuir Isotherm Equation					Freundlich Isotherm Equation			
	*q_e_* (mg/g)	*b* (mg/L)	*R_L_*	R^2^	*p*	*1/n*	*k_f_* (mg/L)	*R*^2^	*p*
103.82	100.76	0.0039	0.1362	0.9793	1.327 × 10^−4^	0.4133	4.614	0.7683	0.0026

**Table 5:** Adsorption/desorption capacities of zinc(II) on XSBLAC after five consecutive cycles.

Recycle Times	1st	2nd	3rd	4th	5th
Adsorption capacity (mg/g)	103.82	94.06	92.62	80.83	42.44
Desorption capacity (mg/g)	78.52	73.31	71.05	60.76	19.17

**Table 6:** Comparison of *q*_max_ for removal of zinc(II) by various adsorbents.

Adsorbent Material	*q*_max_ (mg/g)	Reference
XSBLAC	103.82	This study
SWCNTs	43.66	[46]
MWCNTs	32.68	[46]
PAV	13.04	[46]
Sil/PE1/GA0.5	32.79	[47]
*Frontinalis antipyretica*	14.7	[48]
*PWS*	82	[49]
*Coir*	8.6	[50]
